# Efficacy of a 20:1 CBD:THC cannabis herbal extract for pain and inflammation in dogs following tibial plateau leveling osteotomy

**DOI:** 10.3389/fvets.2025.1676779

**Published:** 2025-09-29

**Authors:** Chloe Lyons, K. Romany Pinto, Kira Penney, Laura Holmes, Abdul Salama, Jane Alcorn, Alan Chicoine

**Affiliations:** ^1^Department of Veterinary Biomedical Sciences, Western College of Veterinary Medicine, University of Saskatchewan, Saskatoon, SK, Canada; ^2^Department of Small Animal Clinical Sciences, Western College of Veterinary Medicine, University of Saskatchewan, Saskatoon, SK, Canada; ^3^College of Pharmacy and Nutrition, University of Saskatchewan, Saskatoon, SK, Canada

**Keywords:** cannabinoids, CBD–cannabidiol, THC–tetrahydrocannabinol, TPLO–tibial plateau leveling osteotomy, cruciate repair, canine

## Abstract

**Objective:**

This study investigates the efficacy of 20:1 cannabidiol:tetrahydrocannabinol (CBD:THC) cannabis herbal extract (CHE) in reducing pain and improving mobility in dogs undergoing tibial plateau leveling osteotomy (TPLO) for cranial cruciate ligament rupture (CCLr).

**Methods:**

Forty-eight dogs were enrolled in a randomized, double-blinded, placebo-controlled clinical trial at the Western College of Veterinary Medicine between December 2022 and October 2024. Dogs were assigned to one of the three treatment groups: placebo (flavored olive oil), 2 mg CBD (0.1 mg THC)/kg body weight (bw), or 5 mg CBD (0.25 mg THC)/kg bw. All dogs received a standard peri- and post-operative analgesic protocol consisting of opioids, nerve blocks, non-steroidal anti-inflammatory drugs (NSAIDs), and gabapentin. Veterinary assessments were performed on days +1 and +14 postoperatively and included Glasgow Composite Pain Scores (GCPS), stifle range of motion (ROM), thigh and stifle circumference, and gait symmetry ratios. Owners were advised to complete a modified Canine Brief Pain Inventory (CBPI) on days +3, +7, and +14. Plasma cannabinoid concentrations on days 1 and 14 were analyzed using liquid chromatography–tandem mass spectrometry (LC–MS/MS). Data were analyzed using linear mixed models and generalized estimating equations.

**Results:**

Forty-two dogs completed the study. All dogs had marked improvement in owner-reported pain scores from days +1 to +14, regardless of treatment group. There were no significant differences in outcome measures between treatment groups. Potential treatment × day interactions were noted for ROM and gait symmetry; the high CHE dose group had marginally greater function on day +1, but none of these effects persisted to day +14. No serious adverse events were reported; mild gastrointestinal effects (vomiting or diarrhea) were noted in seven cases. Owners reported the CHE doses were generally well tolerated. Plasma cannabinoid concentrations were highly variable and did not correlate with clinical outcomes.

**Clinical significance:**

Overall, administration of an oral 20:1 CBD: THC cannabis herbal extract in addition to a standard analgesic protocol did not improve analgesia and limb function following TPLO surgery in dogs. Minor functional benefits may have occurred in the higher CHE dose group, but only on the first day following surgery. Current multimodal peri- and post-operative analgesic practices were effective, irrespective of CHE administration.

## Introduction

Pain is a response to a complex series of internal processes that can be enhanced and diminished by both external and internal sources. Acute pain associated with an external stimulus typically dissipates once the injury resolves; however, during this time, an animal may display behavioral and physiological changes (1). To achieve the highest level of analgesia and reduce the risks of adverse events, multiple drugs targeting various receptors may be required (2, 3).

The endocannabinoid system is a complex regulatory network involved in homeostasis, mood, immune modulation, and pain perception (4–6). Its primary receptors, cannabinoid receptor 1 (CB1) and receptor 2 (CB2), are G-protein-coupled receptors found throughout the nervous and immune systems (4, 6). Activation of these receptors modulates intracellular signaling cascades that reduce neurotransmitter and pro-inflammatory mediator release, attenuating pain and inflammatory pathways (4, 7). Exogenous cannabinoids, particularly tetrahydrocannabinol (THC) and cannabidiol (CBD), interact with this system and are of high therapeutic interest (4, 6).

THC acts as a partial agonist of both CB1 and CB2 receptors and results in the reduction of neuronal excitability (4, 6). In contrast, CBD exhibits more complex and indirect activity, potentially increasing endogenous cannabinoid concentrations through the inhibition of endocannabinoid metabolizing enzymes and reducing receptor desensitization (8, 9). The pharmacokinetics and pharmacodynamics of these cannabinoids can be influenced by the route of administration, formulation, and metabolic interactions, particularly when administered together. This has led to the hypothesis of an “entourage effect,” a term describing combinations of cannabinoids that may alter the pharmacodynamics and/or pharmacokinetics of a single cannabinoid (10, 11).

Pharmacokinetic studies with CBD in dogs suggest low oral bioavailability, with wide variation in plasma concentrations reported (12–20). Such differences may be due to differences in cannabinoid doses, formulations, feeding status, or study populations. Cannabinoids undergo mainly hepatic metabolism, and numerous studies have demonstrated increased liver enzymes such as alanine aminotransferase (ALT) and alkaline phosphatase (ALP) following oral cannabinoid administration (13, 21–23). The overall duration, severity, and clinical implications of such elevated liver enzymes following cannabinoid administration in dogs are unclear. The production of specific cannabinoid metabolites also varies between species, with dogs producing relatively more 6-OH-CBD and 11-OH-THC vs. 7-OH-CBD and 7-COOH-THC as in humans (12, 15, 24).

In veterinary medicine, most evidence for cannabinoid analgesia comes from studies on chronic pain, particularly osteoarthritis in dogs and horses, where several randomized controlled trials have reported beneficial effects using CBD-based treatments (13, 25–30). However, data supporting their use for acute post-operative pain remain limited. One placebo-controlled trial using a CBD/CBDA formulation following tibial plateau leveling osteotomy (TPLO) surgery in dogs reported no significant analgesic or bone-healing benefits (22). Few of the clinical efficacy studies directly reported plasma cannabinoid concentrations alongside observed outcomes, as necessary to develop optimal dosing regimens and therapeutic ranges for cannabinoids.

With limited placebo-controlled studies available, further investigation into cannabinoids’ role in acute pain is needed. The primary objective of this study was to evaluate the analgesic and anti-inflammatory efficacy of a 20:1 CBD: THC cannabis herbal extract (CHE) compared to placebo in dogs undergoing TPLO surgery. We hypothesized that dogs receiving CHE in addition to standard analgesia would demonstrate greater acute pain relief and reduced inflammation. A secondary objective was to explore the relationship between plasma cannabinoid concentrations and clinical outcomes to establish a potential pharmacokinetic-pharmacodynamic (PK-PD) model.

## Methods

This randomized, blinded, placebo-controlled clinical trial was conducted at the Veterinary Medical Center (VMC) of the Western College of Veterinary Medicine (WCVM) between December 2022 and October 2024. Owner consent was obtained prior to enrolment and the study was approved by the University of Saskatchewan’s Animal Research Ethics Board and complied with the Canadian Council on Animal Care. An Experimental Studies Certificate granted by Health Canada’s Veterinary Drugs Directorate permitted the use of cannabinoids for the therapy of client-owned dogs in this study.

*Case inclusion*: Forty-eight canine cases undergoing tibial plateau leveling osteotomy (TPLO) for cranial cruciate ligament rupture (CCLr) were enrolled. Enrollment was based on a power analysis (*β* = 0.80, *α* = 0.05) targeting an effect size of 5–20%, requiring a projected sample size of 60 dogs per treatment group. Tentative diagnosis of CCLr was based on orthopedic examination and radiographic examination by a board-certified veterinary radiologist and was confirmed by arthroscopy during surgery. Inclusion criteria included any dog deemed healthy enough to receive surgery, including any age, sex, breed, body weight, or body condition. Dogs with bilateral CCLr or concurrent orthopedic conditions were also included but analyzed as a separate subpopulation during statistical analysis.

*Surgical procedure and concomitant therapies*: All surgical procedures were performed by ACVS-certified surgeons or supervised by surgical residents at the VMC. Dogs underwent arthroscopy of the affected stifle joint and debridement of any meniscal tear (if present), followed by a TPLO procedure. Postoperative radiographs were obtained to confirm tibial rotation and appropriate implant placement. All dogs received a multimodal peri- and post-operative analgesic regimen, consisting of opioids, femoral and sciatic nerve blocks, non-steroidal anti-inflammatory drugs, gabapentin, and trazodone (Table 1). Dogs were hospitalized overnight following surgery and discharged the following afternoon.

**Table 1:** Summary of analgesic protocol for all dogs undergoing TPLO.

Medical protocol	Medications administered
Preoperative/perioperative Day 0	Sedation: Dexmedetomidine or acepromazine Opioid analgesic: Methadone or hydromorphone Induction: Alfaxalone or ketamine Femoral/sciatic nerve block: Bupivacaine
Postoperative (in clinic) Day +1	Opioid: Methadone or hydromorphone (PRN) NSAIDs: Meloxicam, carprofen, or robenacoxib (as per label dose) Gabapentin (10 mg/kg PO) Trazodone (5 mg/kg PO)
Postoperative (home) Days +1 to +7	NSAIDs (meloxicam or carprofen or robenacoxib, as per label dose) Gabapentin, 10 mg/kg PO, BID or TID

The exact combination of drugs used differed depending on the clinician’s preference. PRN, as necessary; BID, twice a day; TID, thrice a day; PO, orally.

*Treatment allocation*: Cases were randomly assigned to one of the three CHE dose groups via a random number generator (Microsoft Excel) upon time of surgery: placebo (0 mg CBD + 0 mg THC/kg bw), low (2 mg CBD + 0.1 mg THC/kg bw), or high (5 mg CBD + 0.25 mg THC/kg bw). Groups were not blocked by body weight, sex, or other factors. Dog owners and all investigators but one (AC) were blinded to treatment allocation.

*Test item*: CBD-enriched *Cannabis* herbal extract (CHE) with nominal concentrations of 20 mg CBD and 1 mg THC per mL olive oil base (CanniMed) was provided from a licensed cannabis producer (Aurora Cannabis Inc.). This formulation contained no CBG or CBGN. Certificates of analysis were submitted for both CanniMed batches used in the study (Table 2). The placebo was composed of NF-grade olive oil. For all treatments, oil-miscible chicken or beef liver flavoring (PCCA, London, ON, Canada) was added at 3% v/v to improve palatability and facilitate owner blinding by masking the test article scent. Solutions were stored in plastic amber vials and secured in sealed containers to prevent accidental ingestion.

**Table 2:** Cannabinoids and their precursors (CBDA and THCA) found in the CHE treatments.

Cannabinoid	Batch 1 (mg/mL)	Batch 2 (mg/mL)
CBD	19.5	19.8
CBDA	<0.05	0.2
THC	0.9	1.4
THCA	<0.05	0.0

CBD, cannabidiol; CBDA, cannabidiolic acid; THC, tetrahydrocannabinol; THCA, tetrahydrocannabinolic acid; CHE, cannabis herbal extract. Batch 1 was used for cases 1–7, and batch 2 was used for all remaining cases.

*Oral dosing*: Dosing volumes were calculated based on each dog’s body weight, measured the day prior to surgery. The low CHE dose consisted of 2 mg CBD (0.1 mg THC) per kg bw, and the high CHE dose consisted of 5 mg CBD (0.25 mg THC) per kg bw. Placebo olive oil was administered in volumes equivalent to the low CHE dose volume. The first dose was administered the morning after surgery (day +1) by the sole unblinded investigator (AC). This dose was typically administered in the fasting state, as few dogs had consumed canned dog food offered in the morning prior to CHE dosing. Following discharge in the afternoon of D + 1, owners continued CHE administration at home, administered twice daily for 14 days or until recheck. Owners documented the timing and fed/fasting status of each dose. The volume of CHE or placebo administered per dose ranged from 1.6 to 9.4 mL.

*Veterinary measurements*: A summary of the timeline of outcome measures assessed is shown in Table 3. Clinical assessments were conducted on days +1 and +14 by a veterinarian specializing in rehabilitation medicine (KRP and KP) or an investigator trained by these veterinarians (CL). All investigators performing measurements were blinded to the treatment allocation. The same investigator performed all veterinary assessments on the same day, and whenever possible, the same investigator also performed assessments on both days +1 and +14 for the same case. All day +1 veterinary assessments were performed within 1–2 h of initial CHE/placebo treatment. Prior to day +1 veterinary measurements, each dog was assessed for a persistent nerve block represented by a proprioception deficit (lack of knuckling response on the surgical limb). The time between final treatment by the owner and day + 14 veterinary assessment was recorded but varied depending on appointment timing. Veterinary assessment of pain was evaluated using the short form of the Glasgow Composite Pain Scale (GCPS-SF; Supplementary Figure S1) and performed prior to moving the dog from the kennel room to the rehabilitation area.

**Table 3:** Timeline of outcome measure collection over the duration of the study.

Preoperative (day −1)	Postoperative			
	Day +1*	Day +3	Day +7	Day +14
Enrollment Kinetic measurements	Kinetic measurements Circumference measurements Goniometry measurements GCPS Plasma collection	CBPI	CBPI	Kinetic measurements Circumference measurements Goniometry measurements GCPS CBPI Plasma collection

*Measurements performed 1–2 h post-CHE dose. CBPI, Canine Brief Pain inventory; GCPS, Glasgow Composite Pain Scale.

Joint function was assessed by goniometric measurement (angles of flexion and extension) of the surgical stifle. The center of the goniometer was positioned at the center of the stifle joint with one arm aligned along the femur toward the greater trochanter, and the other arm along the tibia toward the lateral malleolus. To measure maximum flexion, maintaining the center and ends of the goniometer fixed to the dog, the tarsus, stifle, and hip were flexed, and the minimum stifle angle was recorded. To measure maximum extension, the goniometer remained fixed in the same position, and the hip, stifle, and tarsus were extended, and the maximum stifle angle was noted. Measurements were taken at an angle where gentle resistance was felt or where any signs of discomfort (vocalization, flinching, biting attempts) were noted. Range of motion (ROM) was calculated as the difference between extension and flexion angles.

Limb inflammation was assessed via thigh and stifle circumference as measured with a flexible tape measure. The thigh circumference was measured at the midpoint between the patella and the greater trochanter. The tape measure was wrapped firmly but not taut around this point and was measured in centimeters to one decimal place. Stifle circumference was measured using the same tape measure, wrapped around the stifle, at the center point of the patellar ligament.

Kinetic assessment was performed using a calibrated pressure-mapping, force-measurement, and tactile sensor walkway (Tekscan 7,101 QL, Tekscan, Inc., Norwood, MA, United States) as used in previously published canine studies (27, 31). Calibration was performed using a step calibration method by a person. Dogs were guided along the sensor in both directions by a single handler until five valid runs were collected (defined as the dog walking at a steady velocity, with no pulling, turning, or head movement, and no steps off the walkway). Dogs were allowed to walk at their preferred walking speed. A variety of kinetic and temporospatial parameters were recorded. Due to a lack of standardization in velocity between days and between dogs, as well as difficulty in determining acceleration due to small numbers of footfalls on the walkway in large dogs, we chose to analyze the ratio of the average peak vertical force (PVF) on the surgical limb compared to the non-surgical limb, rather than absolute values of PVF.

*Owner pain scores*: On days +3, +7, and +14, owners completed a modified version of the Canine Brief Pain Inventory (CBPI; Supplementary Figure S2). As per the original validated CBPI (32), metrics of pain severity and interference relevant to the post-surgical condition were assessed. However, the original CBPI was designed and validated to evaluate pain due to osteoarthritis evaluation, and therefore some questions (e.g., “climbing stairs”) were inappropriate for these patients, given discharge instructions from surgeons. Adverse events, dosage changes, and other observations were also recorded by the owners.

*Plasma cannabinoid concentrations*: Blood samples (2–3 mL) were collected 1.5–2 h after CHE dosing on day +1. Another blood collection was performed on day +14, but due to differences in appointment timing and when the final CHE dose was administered by owners, the time from dose to blood collection varied considerably. Samples were drawn from the cephalic or saphenous veins, centrifuged at 3,500 rpm for 10 min, and plasma was frozen at −70°C. Sample preparation included protein precipitation and lipid removal with Agilent Captiva EMR-Lipid plates. Parent cannabinoid (CBD, THC) concentrations were determined for all plasma samples, with metabolites 6-OH-CBD, 7-OH-CBD, 7-COOH-CBD, 11-OH-THC, and 11-COOH-THC performed on a subset of samples. Concentrations were quantified using an LC–MS/MS method (Agilent 1,290 Infinity HPLC coupled with a SCIEX QTrap® 6,500 mass spectrometer using a Zorbax Eclipse XDB-C18 column) previously validated for canine plasma (12, 33).

### Statistical analysis

Statistical analyses were conducted with commercially available software (SPSS v28, Chicago, IL, United States). Normality of continuous outcomes was assessed using the Shapiro–Wilk test and visual inspection of residuals. Descriptive statistics are presented as mean (95% confidence interval) for normally distributed outcomes and median (interquartile range) for non-normally distributed outcomes.

Linear mixed models (LMMs) were used for normally distributed data (ROM, stifle circumference, symmetry ratios), while non-parametric continuous outcomes (flexion, thigh circumference) were analyzed using generalized estimating equations (GEEs). CBPI and GCPS scores, treated as count data, were analyzed using Poisson GEE models. Significance was set at *α* = 0.05, with a Bonferroni-adjusted significance threshold of α = 0.025 applied to the flexion and extension analyses, as they were included in the ROM analysis.

Fixed effects in the models included the CHE treatment group (dose), time point (day), and their interaction. Additional covariates assessed in backward selection included body weight, age group (>5 vs. ≤5 years), sex, bilateral/unilateral CCLr status, and presence/absence of meniscal tear. Due to potential confounders, some cases were excluded from statistical analysis for specific outcome measures. Dogs with persistent day +1 nerve blocks were excluded from ROM, GCPS, and symmetry analysis on day +1 due to inability to resist manipulation or body weight-bearing or feel pain around the affected joint and incision site. Dogs with bilateral CCLr at the time of (unilateral) TPLO surgery were excluded from symmetry ratio analysis due to expected skewed body weight-bearing. The body condition score was excluded from analysis due to its non-normal distribution across groups.

Differences in cannabinoid concentrations between low and high CHE dose groups on day +1 were analyzed using a Mann–Whitney U-test. Due to the inconsistent timing of blood collection relative to CHE administration on day +14, only a descriptive analysis of plasma concentrations is presented for this day.

## Results

### Patient demographics

Forty-eight cases were initially enrolled, with 42 cases included in the final analysis. Six cases were excluded from the final analysis for the following reasons: lack of follow-up visit (*n* = 2, both in the placebo group), dog too aggressive to perform physical outcome measures following surgery (*n* = 1, low-dose CHE group), owner did not pick up study medication (*n* = 1, high-dose CHE group), owner could not perform oral dosing at home (*n* = 1, high-dose CHE group), and additional orthopedic surgical procedure performed on the same day of TPLO surgery (*n* = 1, placebo group). Of note, data from 42 surgical cases included in the final analysis were collected from 37 dogs. Five dogs in the study population required TPLO surgery on both hind limbs over the 2-year study duration, with each surgery counted as a separate case.

A summary of relevant patient characteristics is shown in Table 4, with individual case data available in Supplementary Table S1. Among the 42 cases that completed the study, 18 were male (14 castrated and four intact), and 24 were female (23 spayed and one intact). Case age ranged from 3 to 11 years (median = 6 years), with a body weight range of 14.5–52 kg (median = 31.9 kg) and a body condition score range (1–9 scale) of 4–8 (median = 6). There was a statistically significant difference in body weight between the dosage groups (*p* = 0.01, Kruskal–Wallis ANOVA), with the 2 mg CBD/kg (low dose) group having a higher body weight rank than the placebo group. There were no differences in age (*p* = 0.72) or body condition score (*p* = 0.14) between dosage groups. The most common breeds were German Shepherd/crosses (*n* = 11 cases), Labrador/crosses (*n* = 9 cases), and Husky/crosses (*n* = 4 cases). Of the TPLO surgeries, 24 were performed on the right hind limb and 18 on the left. Complete cruciate ligament rupture was diagnosed in 24 cases, with 11 cases having partial tears and 7 having an unknown status. Upon surgical examination, 28 cases were also diagnosed with meniscal tears. During the study period, a total of 10 cases had bilateral CCLr (both ligaments torn and unrepaired at the time of TPLO surgery). Other non-stifle musculoskeletal abnormalities were noted in five cases by the surgeon or rehabilitation veterinarian during the pre-operative orthopedic exam (including carpal valgus, ulnar nerve damage, and bilateral hip dysplasia). As none of the concurrent abnormalities were considered sufficient to bias the post-TPLO outcome measures, these cases were included in the final analysis.

**Table 4:** Descriptive statistics (median and range) of cases summarized by the treatment group.

Treatment	Body weight (kg)	BCS^4^	Age (years)	Sex ratio (Male: Female)	Complete vs. partial cruciate ligament tears	Meniscal tear (yes/no)	Bilateral CCLr^5^ (yes/no)
Overall study (*n* = 42)^1^	31.9 (15.4–52.0)	6 (4–8)	6 (3–11)	4 intact males 14 neutered males 1 intact female 23 spayed females	24 complete 11 partials 7 unknowns	28 yes 14 no	10 yes 32 no
Placebo (*n* = 16)	31.1^A^ (15.4–43.6)	6 (4–8)	7 (3–11)	2 intact males 4 neutered males 1 intact female 9 spayed females	11 complete 3 partial 2 unknown	12 yes 4 no	5 yes 11 no
Low-dose CHE^2^ (*n* = 13)	36.6^B^ (22.1–52.0)	6 (5–8)	6 (4–9)	1 intact male 6 neutered males 6 spayed females	4 complete 5 partial 4 unknown	11 yes 2 no	1 yes 12 no
High-dose CHE^3^ (*n* = 13)	30.2^AB^ (22.3–37.6)	5 (5–7)	5 (4–11)	1 intact male 4 neutered males 8 spayed females	9 complete 3 partial 1 unknown	5 yes 8 no	4 yes 8 no

1 Number of cases completing the study.

2 Low dose CHE = 2 mg CBD (0.1 mg THC)/kg bw.

3 High dose CHE = 5 mg BCD (0.25 mg THC)/kg.

4 BCS, body condition score (1–9 scale), as determined by a rehabilitation veterinarian.

5 Both cranial cruciate ligaments were ruptured and unrepaired at the time of TPLO surgery.

Statistically significant differences between groups are denoted by alphabetical superscript (*p* < 0.05, Kruskal–Wallis ANOVA with Dunn’s multiple comparisons test).

### Veterinary assessments

At the time of initial outcome assessment (1–2 h following CBD/placebo treatment in the morning following TPLO surgery), 10 cases demonstrated signs consistent with ongoing femoral/sciatic nerve block (lack of knuckling response on the surgical limb). These cases were removed from the GCPS, goniometry, and kinetic analyses, as the lack of pain sensation in the limb or resistance to limb manipulation would confound these results.

Veterinary pain score: Glasgow Composite Pain Scores (GCPS) were generally low (minimal pain) on day +1, with median scores of 2, 3, and 3 for the placebo, low, and high CHE dose groups, respectively. The median GCPS score on day +14 was 1 for all treatment groups. The analysis of GCPS using a general estimating equation model revealed a significant main effect of day (*p* = 0.004, day +1 higher than day +14) but not of dose (*p* = 0.990). Additionally, sex was a significant factor, with female dogs displaying higher GCPS scores than males (*p* < 0.001).

**Figure 1:**
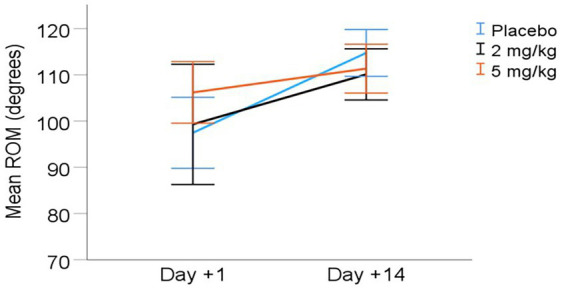
Mean (± 95% CI) range of motion (ROM) from day +1 to day +14 post-TPLO. ROM calculated as the difference between angle of stifle extension and flexion (degrees).

Goniometry: The stifle range of motion (ROM) increased significantly between days +1 and +14 (overall mean increase of 11.1° from days +1 to +14 for all treatment groups combined, *p* < 0.001). See Table 5 and Figure 1 for ROM values. However, there were no statistically significant differences in ROM between treatment groups (*p* = 0.420). A dose × day interaction for ROM approached significance (*p* = 0.057), with the 5 mg/kg group approaching a higher ROM than other treatments at D + 1.

**Table 5:** Veterinary-assessed outcome measures for range of motion (ROM), limb circumference, and hind limb symmetry ratios on days +1 and +14 following TPLO surgery.

Outcome measure	Treatment group	Day +1	Day +14	Difference (D + 14–D + 1)
Range of motion (ROM, °) *Mean (95% CI)*	Placebo	97.4° (89.8–105.1)	114.7° (109.7–119.8)	+17.3° *
	Low-dose CHE	99.3° (86.3–112.3)	110.1° (104.6–115.6)	+10.8° *
	High-dose CHE	106.2° (99.5–112.8)	111.3° (106.1–116.6)	+5.1° *
	**All Groups Combined**	101.2° (96.7–105.7)	112.3° (109.5–115.2)	+11.1° *
	Dose × Day: p = 0.057			
Stifle Circumference (cm) *Mean (95% CI)*	Placebo	27.4 cm (25.4–29.3)	23.2 cm (20.9–25.5)	−4.2 cm *
	Low-dose CHE	31.7 cm (28.3–35.1)	28.2 cm (26.8–29.6)	−3.5 cm *
	High-dose CHE	27.9 cm (26.8–29.1)	25.1 cm (23.7–26.4)	−2.8 cm *
	**All Groups Combined**	28.8 cm (27.5–30.2)	25.3 cm (24.0–26.5)	−3.5 cm *
Thigh Circumference (cm) *Median (IQR)*	Placebo	30.5 cm (5.6)	28.5 cm (6.5)	−2.0 cm *
	Low-dose CHE	34.0 cm (12.0)	32.0 cm (5.1)	−2.0 cm *
	High-dose CHE	32.0 cm (5.0)	30.8 cm (3.8)	−1.2 cm *
	**All Groups Combined**	32.5 cm (6.4)	31.0 cm (4.3)	−1.5 cm *
Hind Limb Symmetry Ratio *Mean (95% CI)*	Placebo	0.49 (0.25–0.73)	0.73 (0.63–0.83)	+0.24
	Low-dose CHE	0.59 (0.26–0.91)	0.58 (0.47–0.69)	−0.01
	High-dose CHE	0.74 (0.55–0.93)	0.63 (0.47–0.79)	−0.11
	**All Groups Combined**	0.59 (0.46–0.72)	0.65 (0.59–0.71)	+0.06
	Day × Dose: p = 0.035 *			

Mean or median differences reflect the change from days +1 to +14 within each group. Range of motion is determined from the maximum angle of extension (degrees) minus the minimum angle of flexion (degrees). Symmetry was calculated as the mean of the peak vertical force of the surgical/non-surgical hind limb over five runs. Asterisks (*) indicate statistically significant differences between day +1 and day +14 within the same treatment group. Day × Dose interactions are included where relevant. Low dose CHE = 2 mg CBD + 0.1 mg THC/kg bw. High dose CHE = 5 mg CBD + 0.25 mg THC/kg bw.

**Figure 2:**
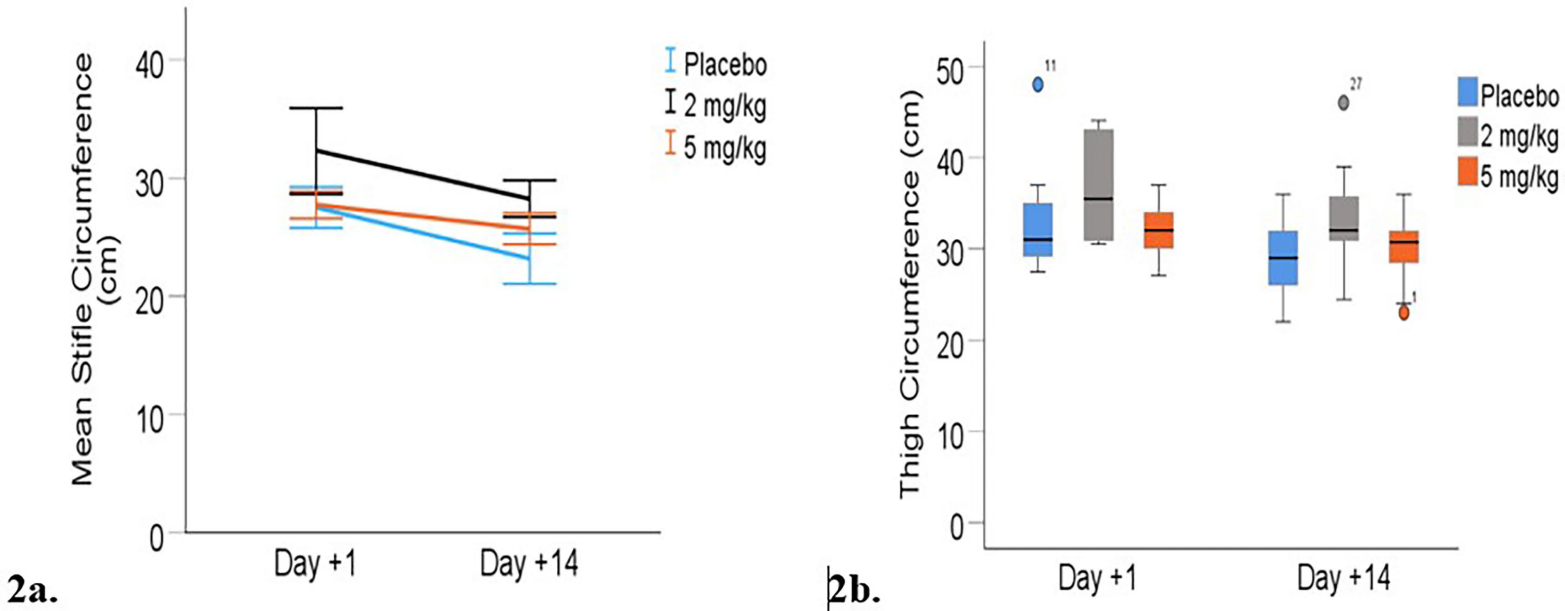
Limb circumferences (cm) from day +1 to day +14 post-TPLO. **(a)** Mean (+/− 95% CI) stifle circumference. **(b)** Median thigh circumferences. Median is displayed by the black line in the boxes the boxes represent interquartile range (IQR and whiskers are the range of data). Mild outliers (defined by being greater than 1.5 times IQR) are represented by circles.

Limb circumference: Heavier dogs had significantly larger limb measurements across time points (*p* < 0.001). The combined mean predicted limb measurements decreased between days +1 and +14 for both stifle and thigh circumference measurements (*p* < 0.001; Table 5). Stifle circumference decreased by an average of 3.5 cm from day +1 to day +14 (*p* < 0.001), while mean thigh circumference decreased by 1.5 cm over the same period (*p* < 0.001), indicating reduced inflammation or possible post-surgical muscle atrophy during the first 2 weeks following surgery. There was no statistically significant effect of the treatment group on either stifle or thigh circumference at either time point (Figure 2).

**Figure 3:**
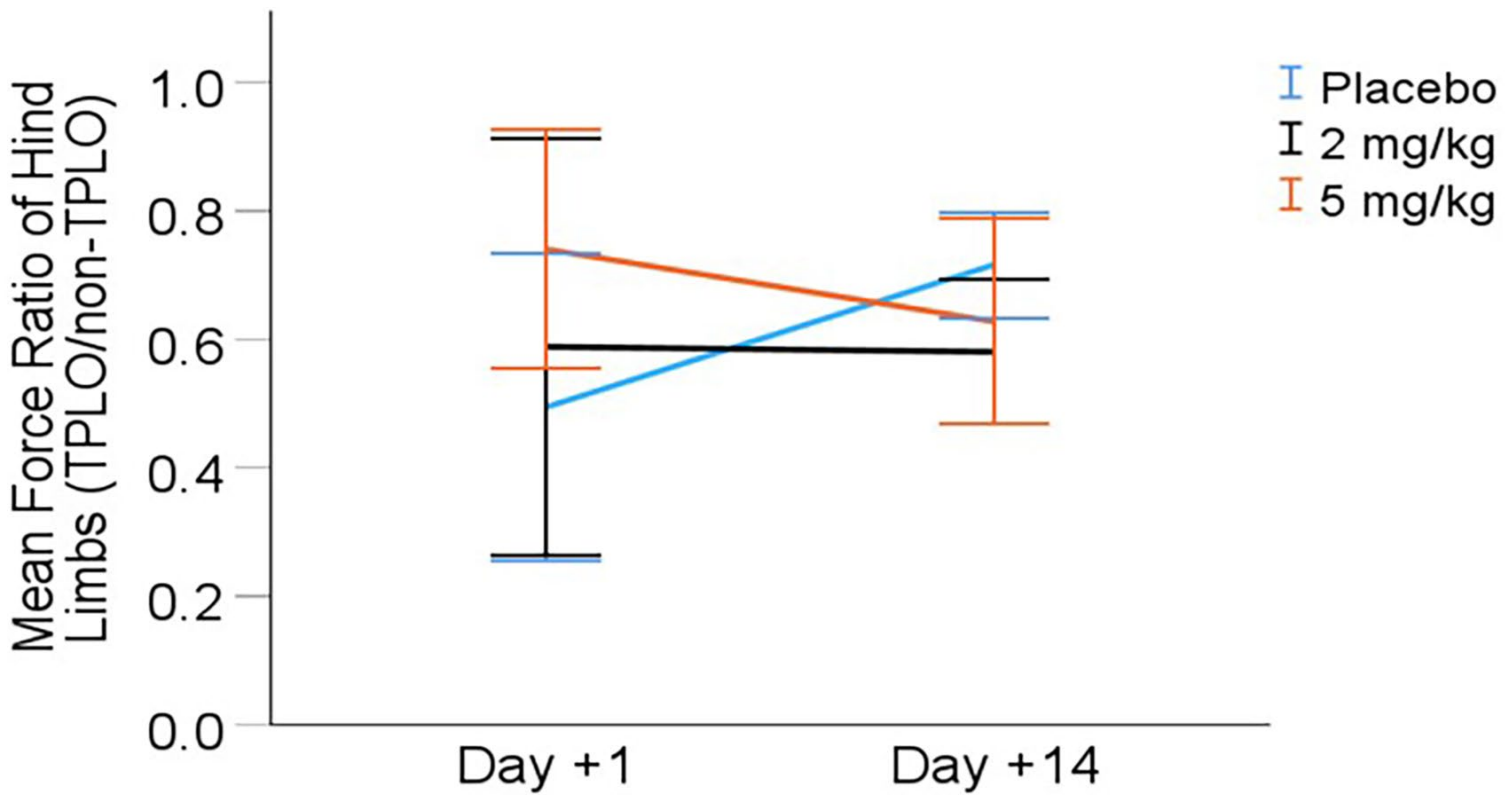
Mean (± 95% CI) symmetry (hind limb force ratio) from day +1 to day +14 post-TPLO (Ratio calculated as PVF of TPLO leg divided by P VF ofnon-TPLO leg).

Kinetic assessment: Symmetry (ratio of peak vertical force production on surgical vs. non-surgical hind limb) results are presented in Table 5 and Figure 3. The final LMM for symmetry included the variables treatment group (dose), day, and treatment × day interaction. There was a significant interaction effect between treatment and day on the symmetry ratio (*p* = 0.035). *Post*-*hoc* comparisons with the Bonferroni correction showed a potential difference between the placebo and 2 mg/kg groups on day +14 (*p* = 0.061), with the placebo group having increased hind limb symmetry (Table 5).

### Owner assessment

**Figure 4:**
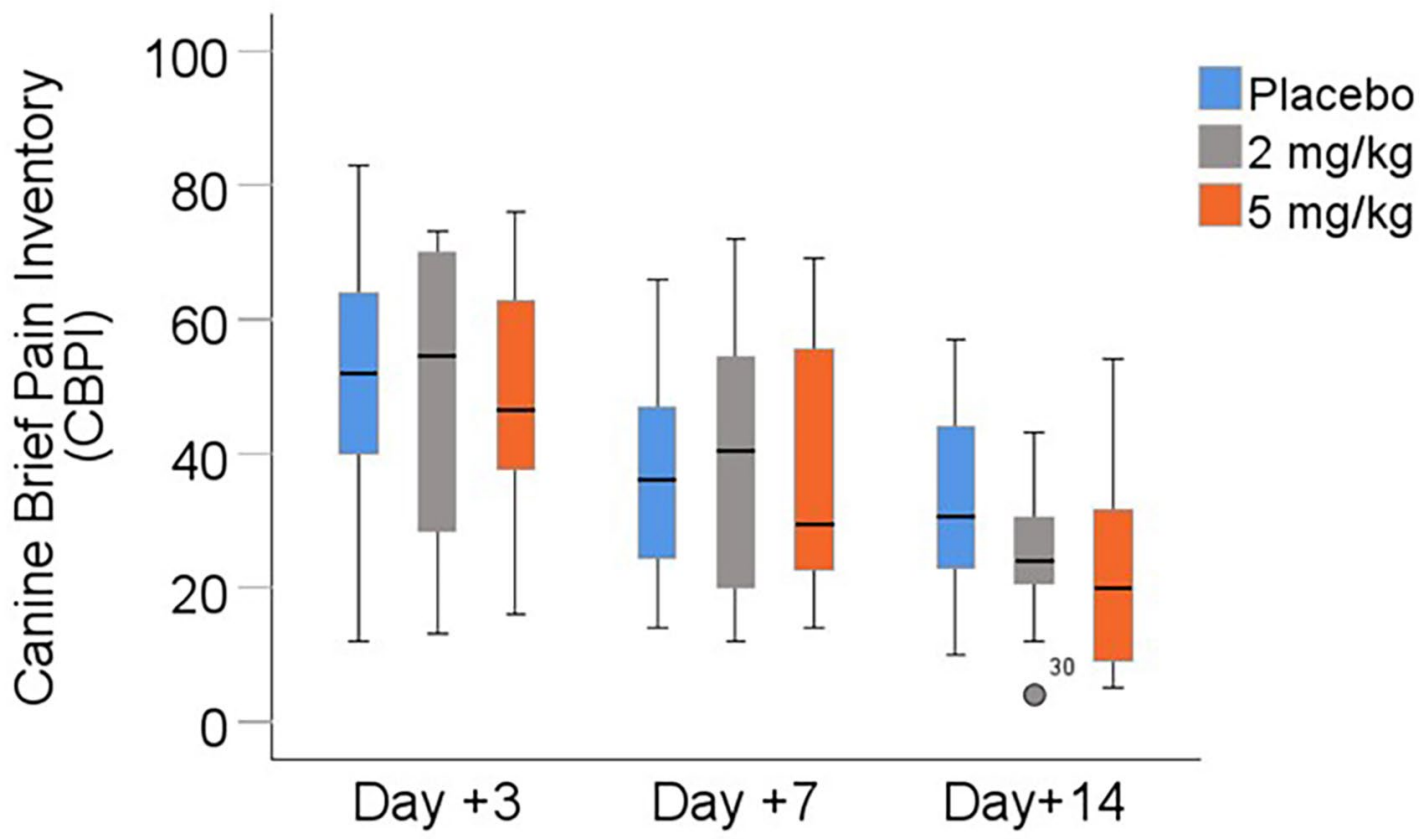
Owner-reported pain scores (modified Canine Brief Pain Inventory) between days +3, +7, and +14 following TPLO surgery. Median is displayed by the black line in the boxes, the boxes represent interquartile range (IQR), and whiskers are the range of data. The open circle represents a mild outlier defined as greater than 1.5 times the IQR. I.

Pain scores assessed via the modified Canine Brief Pain Inventory (CBPI) are shown in Table 6 and Figure 4. There was a statistically significant difference in scores between days following surgery (*p* < 0.001), with all treatment groups demonstrating lower (improved) pain scores from days +3 to +7 to +14. However, the treatment group had no significant effect (*p* > 0.6) on owners’ pain scores.

**Table 6:** Owner-reported pain scores from the modified Canine Brief Pain Inventory (CBPI) on days +3, +7, and +14 following TPLO surgery.

Treatment group	Day +3	Day +7	Day +14	Difference (from day +3 to day+14)
Placebo	52 (26)	36 (27)	31 (31)	−21 *
Low-dose CHE	54 (43)	41 (26)	24 (16)	−30 *
High-dose CHE	47 (27)	30 (34)	20 (31)	−27 *
**All groups**	51 (30)	36 (32)	24 (18)	−27 *

Scores are presented as median (IQR). The final column represents the change in median scores from day +3 to day +14. Asterisks (*) indicate statistically significant reductions in pain scores over time (*p* < 0.05). Low-dose CHE = 2 mg CBD +0.1 mg THC/kg bw. High-dose CHE = 5 mg CBD + 0.25 mg THC/kg bw.

### Adverse events

Over the duration of the study, owners reported a total of 15 adverse events in 14 cases (33.3% of the study population). Twenty-eight cases (66.7%) reported no adverse events. The most commonly reported adverse events were vomiting, diarrhea, and lethargy/somnolence (see Table 7). While not statistically significant, the placebo group had more counts of vomiting, diarrhea, and lethargy than the other treatment groups. No case had an adverse event persist throughout the study, except for one dog (placebo group) whose owner reported frequent eructation throughout the trial, potentially indicating regurgitation of the oil. The most commonly reported adverse events were vomiting, diarrhea, and lethargy/somnolence (see Table 7). Some owners indicated that their dogs increasingly disliked the oil mixture (whether CHE or placebo) after repeated administration, whether administered directly in the mouth or mixed with food. The most serious adverse event reported occurred in a non-study dog that accidentally gained access to a bottle of CHE and consumed up to 80 mL. Clinical signs included vomiting, lethargy, and ataxia, which resolved within 48 h.

**Table 7:** Counts of the most common adverse events reported by owners over the study duration.

Adverse event	Treatment group	Day +3	Day +7	Day +14	Overall study
Vomiting	Placebo	1	0	2	3
	Low-dose CHE	1	0	0	1
	High-dose CHE	0	0	0	0
	All groups	2	0	2	4
Diarrhea	Placebo	1	0	1	2
	Low-dose CHE	0	0	0	0
	High-dose CHE	0	1	0	1
	All groups	1	1	1	3
Lethargy	Placebo	4	0	0	4
	Low-dose CHE	1	0	0	1
	High-dose CHE	2	0	0	2
	All groups	7	0	0	7

*n* = 16 (placebo), *n* = 13 (low dose CHE), *n* = 13 (high dose CHE). Low-dose CHE = 2 mg CBD +0.1 mg THC/kg bw. High-dose CHE = 5 mg CBD + 0.25 mg THC/kg bw.

### Plasma cannabinoid concentrations

**Figure 5:**
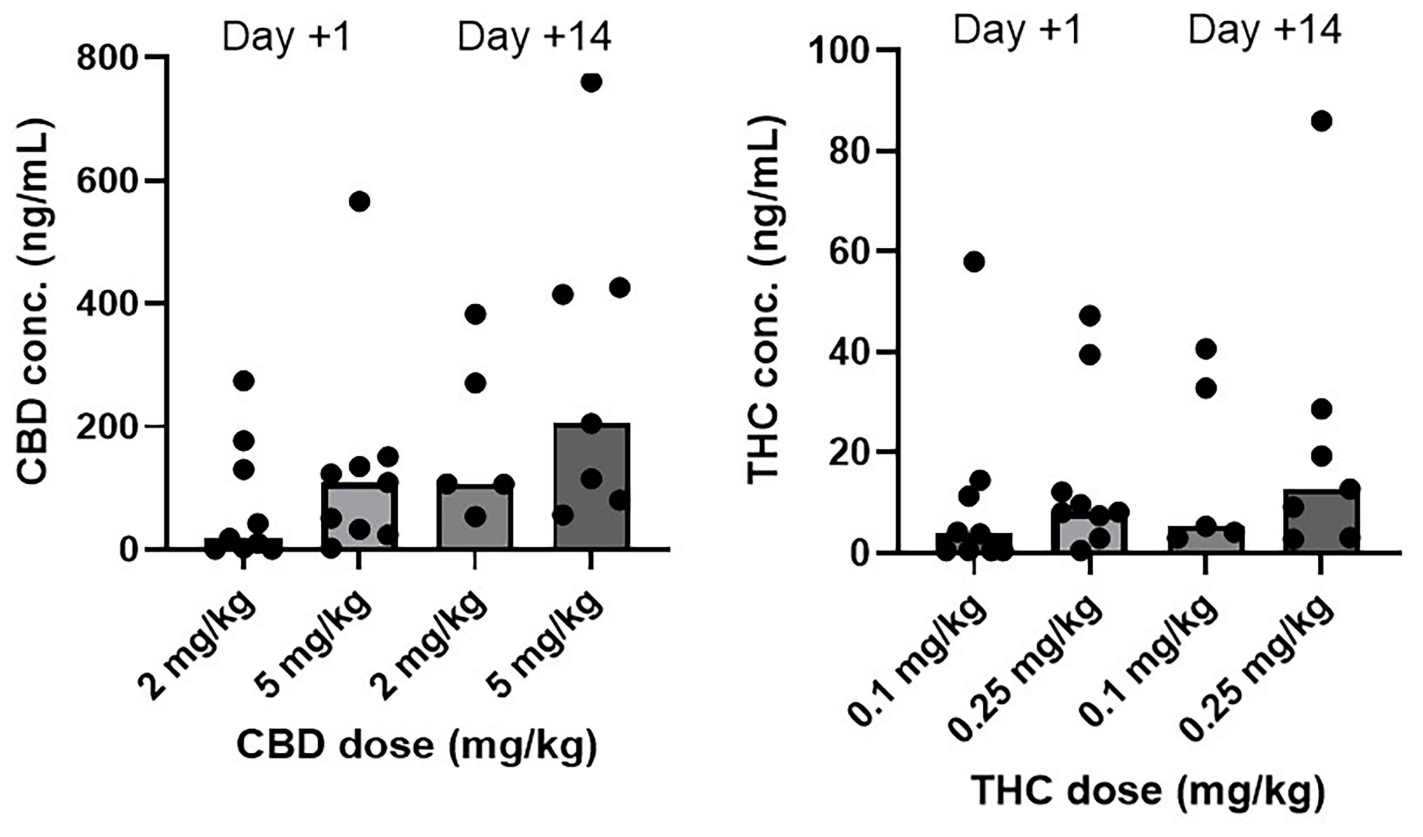
CBD and THC plasma concentrations (ng/mL) by dose group and day. Median is displayed by the top of boxes. Day +1 plasma sample collected 1.5–2 h following initial CHE dose. Day +14 plasma sample collected at variable time following final CHE dose. All cannabinoid plasma concentrations from placebo treated dogs were <LOD (0.5 ng/mL).

The concentrations of CBD and THC from CHE-treated dogs are depicted in Table 8 and Figure 5. Concentrations for individual dogs, along with a subset of samples analyzed for cannabinoid metabolites, are shown in Supplementary Tables S2, S3. No cannabinoids were detected in the plasma of any placebo-treated dogs. There were no statistically significant differences in cannabinoid concentrations between the low, high, and CHE dose groups on day +1 (*p* = 0.287 for CBD, *p* = 0.291 for THC, Mann–Whitney U-test). Statistical comparisons incorporating day +14 concentrations were not deemed appropriate due to the substantial variance in the timing of sample collection relative to the final dose between cases.

**Table 8:** Summary of CBD and THC plasma concentrations (ng/mL) on days +1 (n = 19) and +14 (*n* = 11).

Cannabinoid	Day	Dose (mg/kg bw)	Median (ng/mL)	Minimum (ng/mL)	Maximum (ng/mL)
CBD	+1	2	18.5	<LOD	274
		5	109	<LOQ	566
	+14	2	107	53.7	383
		5	205	56.2	761
THC	+1	0.1	3.8	<LOQ	58.0
		0.25	8.2	<LOQ	47.3
	+14	0.1	5.4	3.0	40.7
		0.25	12.8	2.8	86.0

Limit of quantification (LOQ) = 2.0 ng/mL. Limit of detection (LOD) = 0.5 ng/mL.

## Discussion

It was hypothesized that the inclusion of an oral cannabis herbal extract (CHE) following TPLO surgery in dogs would reduce post-operative pain and inflammation in a dose-dependent manner. While individual cases demonstrated significant variability in specific outcomes measured, all cases (including CHE-treated and placebo groups) demonstrated remarkable clinical improvement over the 14-day study duration. As there were no clinically or statistically significant differences in day +14 outcomes, this study did not demonstrate evidence to justify the inclusion of oral CHE as part of an otherwise robust post-TPLO analgesic regimen.

However, it is possible that high-dose CHE administration immediately following surgery provided a short-term benefit. Range of motion (ROM) showed a day × dose trend approaching statistical significance (*p* = 0.071), with the high CHE dose group potentially exhibiting greater ROM on day +1 compared to the other treatment groups. The higher CHE dose group also appeared to have the highest symmetry of hind limb force production on day +1, though this finding was not statistically significant. Potential improvements in ROM and/or hind limb force symmetry in the high CHE dose group on day +1 could hypothetically be due to additional analgesia or sedation early in the postoperative period. However, the small sample size of the treatment groups and high variance in outcomes limited the power to detect such potential differences with statistical significance. Any possible benefit of high CHE administration on D + 1 was not observed on day +14 (owner or veterinarian pain scores, goniometry, limb measurements, or kinetic assessment). In the absence of additional measurements, the duration of the potential benefit of high CHE administration following surgery is unknown.

Previous cannabinoid studies for musculoskeletal conditions in dogs have also used both gait assessments (objective or subjective) alongside subjective pain measures, as was performed in this study. Gamble et al. and Talsma et al. studies both reported reductions in owner-assessed (subjective) pain, but without corresponding statistically significant improvements in objective or subjective gait measures (13, 27). An explanation may be that due to high variance in gait measurements between and within dogs, or due to the potentially limited effect size of cannabinoids on gait measures, any such improvements are difficult to demonstrate.

Among all outcome measures used in this study, owner-reported pain scores (modified CBPI) appeared the most consistent. Owner pain scores indicated improvement from day +3 to +7 to +14 following surgery for all CHE treatment groups. The finding of no significant CHE treatment effects on owner pain scores aligns with a previously published TPLO study that also found no reduction in subjective pain scores with the addition of cannabinoids (22). In contrast, improvements in CBPI have been reported in studies evaluating cannabinoids for chronic osteoarthritis using similar doses and formulations (13, 25, 27). Cannabinoid research in horses with osteoarthritis using a comparable observer pain scoring system has also demonstrated beneficial results (34). This may suggest greater cannabinoid efficacy for chronic rather than acute musculoskeletal pain in dogs, possibly due to differences in the underlying chronic vs. acute pain mechanisms. Alternatively, concurrent analgesics routinely administered in surgical studies may mask potential cannabinoid effects. For example, all dogs in this study received extensive peri-operative and post-operative analgesia, likely limiting any potential additive effects from the CHE.

In a similar study design, Klatzkow et al. also used the same TPLO surgical model to assess the effects of a 2 mg/kg cannabinoid treatment (40:1 CBD:THC) administered BID over 28 days (22). The study also found no significant differences in subjective pain scores between the cannabinoid and placebo groups, and it found no radiographic evidence of enhanced joint healing after 28 days (which was not assessed in this study). Because the previous study used only subjective assessments to evaluate pain, this study incorporated additional objective outcome measures (goniometry, limb force production) to detect potential but subtle changes in acute post-operative pain. Another difference between the studies was the CHE doses used. While the previous study included one CHE treatment group (2 mg CBD/kg bw), the current study utilized this same dose plus a higher dose (5 mg CBD/kg bw) in case dose-dependent CHE effects occurred, as has been observed in some canine osteoarthritis studies demonstrating increased therapeutic effects with higher CHE doses (13, 26).

Plasma cannabinoid concentrations in this study were similar to those reported in other studies of dogs using comparable CHE doses (12, 13, 15, 16, 19). Concentrations on day +1 were highly variable within both CHE dose groups, despite a consistent sampling time in all cases (1.5–2 h following CHE administration, based on the typical time to maximum plasma concentration after oral administration of this CHE product in dogs and cats) (12, 35). Some of the variability may be attributed to the inconsistent feeding status of the dogs at the time of day +1 CHE administration. Although all dogs had been offered canned dog food up to 1 h prior to CHE dosing, most dogs in this early post-operative state had consumed none or a very small amount. Concentrations were also highly variable on day +14, with the most plausible explanation due to vast differences in the time of sample collection relative to the last dose between cases. Some owners did not administer a final CHE dose on the morning of the day +14 assessment, meaning the previous dose was 12–18 h before sample collection. Owner non-compliance with CHE dosing or variable feeding status at the time of dosing is another potential explanation for the day +14 variance observed. The predominant CBD metabolites identified in the analysis of a subset of plasma samples were 6-OH-CBD and 7-COOH-CBD, consistent with the results observed in previous canine studies (12, 15, 24).

Adverse events as reported by owners were mild and consistent with adverse events observed in previous cannabinoid studies following TPLO surgery (22) or using the same CHE formulation in dogs (12). Vomiting, diarrhea, and lethargy were reported as sporadically occurring events across all groups. Of note, lethargy was reported in seven cases on day +3 but reported in any cases on days +7 or +14. As four of the seven instances reporting lethargy were in the placebo group, it is unlikely that these signs were due to CHE administration. Instead, the owner may have interpreted their dog’s slow recovery from major orthopedic surgery as “lethargy” or may have presumed their dog was included in a CHE treatment group and therefore assumed such side effects were occurring. Interestingly, no owner reporting lethargy considered it an “adverse” event. Some owners considered the decreased activity as a positive outcome, as it minimized the activity of their high-strung dog after surgery, which facilitated compliance with the surgeon’s discharge instructions. Some owners in all treatment groups reported that their dog became increasingly reticent to ingest the oral medication when administered BID for 14 days, whether administered directly in the mouth or on food. Despite all treatments containing flavoring agents to improve palatability, it is possible that the volumes of olive oil base administered (up to 9 mL in the high CHE dose group) were too large for the dog to tolerate. The most serious adverse event did not occur in a dog enrolled in the study but rather in a different dog in the same household, which inadvertently gained access to a bottle of CHE and ingested up to 80 mL (approximately 53 mg CBD/kg and 2.6 mg THC/kg). The dog experienced acute vomiting with signs of lethargy and ataxia for the first 12 h after ingestion, with all signs resolving after 2 days. This adverse event in a non-target animal highlights the importance of educating owners about proper storage of cannabinoids in the household. The lack of otherwise serious adverse effects in the 26 CHE-treated dogs suggests this CHE formulation is safe when used at these dose regimens.

The primary limitation of this study was its small sample size, limiting the power to detect potential treatment effects. Power was further reduced by utilizing both low and high CHE dose groups, opposed to one large CHE group. Cases were randomized to treatment group without blocking by dog’s body weight or other parameters (sex, body condition score). With small sample sizes and without blocking treatment allocation by body weight, the low CHE dose group (2 mg CBD/kg bw) randomly received a heavier distribution of dogs than the other dose groups. Body weight is a potential confounder for many of the outcomes assessed (goniometry, limb measurements, and symmetry of hindlimb force). Although the models included body weight in their equations, the unequal distribution of body weights between treatment groups may have potentially masked a dose effect. Inclusion of a robust multimodal analgesic protocol in all cases likely reduced the magnitude of any potential CHE effect. This clinical trial also recruited a broad range of cases, with extensive variability in dog size, body weight, concurrent musculoskeletal abnormalities (e.g., bilateral CCLr and meniscal tear), and timing of CCLr relative to TPLO surgery. Ensuring an even distribution of cases with such factors between the three treatment groups would have limited the potential for biasing the study results. Inconsistent owner compliance with dosing and post-operative restrictions may also have limited the detection of potential cannabinoid treatment effects, as well as the day +14 plasma results.

Overall, this study did not demonstrate that a 20:1 CBD:THC cannabis herbal extract, administered at 2 or 5 mg CBD/kg bw BID for 14 days, provided additional analgesic or anti-inflammatory benefits beyond a standard analgesic protocol in dogs following TPLO surgery. Due to substantial differences in patient characteristics and a relatively small sample size, the study may not have had sufficient power to detect potential therapeutic effects. A mild increase in some limb function measures may have occurred in the high CHE dose group, but only in the immediate postoperative period. All dogs demonstrated significant functional improvement and a decrease in pain scores over the course of the study. Plasma cannabinoid concentrations were variable and did not correlate with clinical outcomes.

## Data Availability

The raw data supporting the conclusions of this article will be made available by the authors, without undue reservation.
