# Supplementary material for: Efficacy of a 20:1 CBD:THC cannabis herbal extract for pain and inflammation in dogs following tibial plateau leveling osteotomy
Source: Front Vet Sci. 2025 Sep 29;12:1676779. doi: 10.3389/fvets.2025.1676779 (PMC12515679; doi:10.3389/fvets.2025.1676779)
Supplement: Supplementary file 1 [file Data_Sheet_1.docx]

**Supplemental Figure 1.** Short form of the Glasgow Composite Pain Scale (GCPS-SF) used by rehabilitation veterinarians to assess pain day +1 and +14 after surgery.


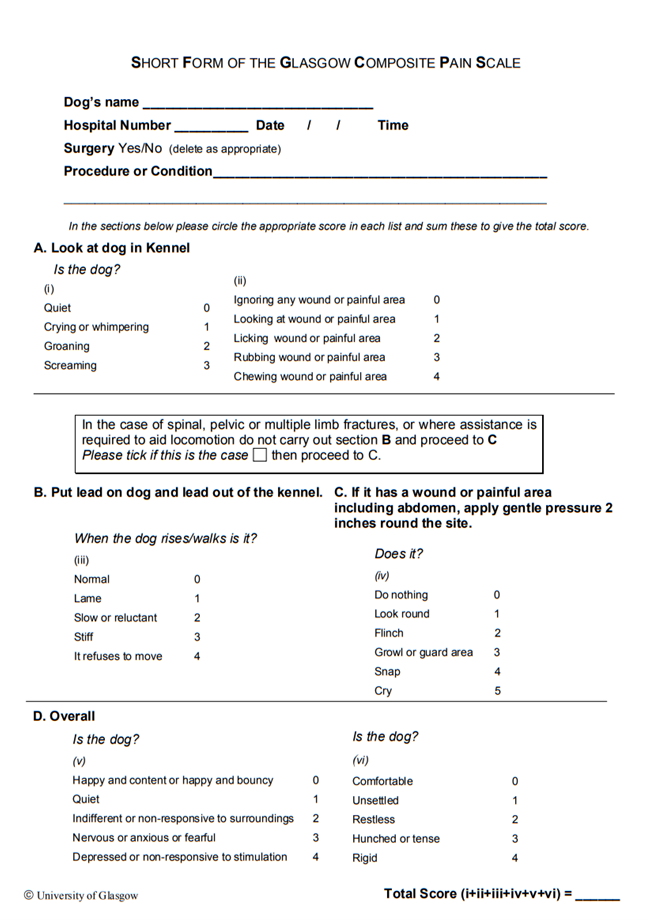


**Supplemental Figure 2.** Example of Modified Canine Brief Pain Inventory (CBPI) performed by owners on days +3, +7, and +14.

***Canine Brief Pain Inventory – 7 days after surgery***

**Description of pain:**

Rate your dog’s pain:

1. Fill in the oval next to the one number that best describes the pain at its ***worst*** in the last ***7 days***.

○0 ○1 ○2 ○3 ○4 ○5 ○6 ○7 ○8 ○9 ○10

No pain Extreme pain

2. Fill in the oval next to the one number that best describes the pain at its ***least*** in the last ***7 days***.

○0 ○1 ○2 ○3 ○4 ○5 ○6 ○7 ○8 ○9 ○10

No pain Extreme pain

3. Fill in the oval next to the one number that best describes the pain at its ***average*** in the last ***7 days***.

○0 ○1 ○2 ○3 ○4 ○5 ○6 ○7 ○8 ○9 ○10

No pain Extreme pain

4. Fill in the oval next to the one number that best describes the pain as is ***right now***.

○0 ○1 ○2 ○3 ○4 ○5 ○6 ○7 ○8 ○9 ○10

No pain Extreme pain

**Description of function:**

Fill in the oval next to the one number that best describes how during the last 7 days pain has interfered with your dog’s:

5. **General Activity** *(within limits specified by surgeon)*

○0 ○1 ○2 ○3 ○4 ○5 ○6 ○7 ○8 ○9 ○10

Does not interfere Completely interferes

6. **Enjoyment of Life**

○0 ○1 ○2 ○3 ○4 ○5 ○6 ○7 ○8 ○9 ○10

Does not interfere Completely interferes

7. **Ability to Rise to Standing From Lying Down**

○0 ○1 ○2 ○3 ○4 ○5 ○6 ○7 ○8 ○9 ○10

Does not interfere Completely interferes

***Brief Pain Inventory, con’t***

8. **Ability to Walk (in general)**

○0 ○1 ○2 ○3 ○4 ○5 ○6 ○7 ○8 ○9 ○10

Does not interfere Completely interferes

9. **Ability to Walk (bearing weight on surgically-repaired hind limb)**

○0 ○1 ○2 ○3 ○4 ○5 ○6 ○7 ○8 ○9 ○10

Does not interfere Completely interferes

10. **Ability to Climb Stairs, Curbs, Doorsteps, etc.** ○Without assistance ○With assistance

○0 ○1 ○2 ○3 ○4 ○5 ○6 ○7 ○8 ○9 ○10

Does not interfere Completely interferes

**Overall impression:**

11. Fill in the oval next to the one number that best describes your dog’s overall quality of life over the last 7 days.

○ Poor ○ Fair ○Good ○Very Good ○Excellent

**Other Comments:**

**Supplemental Table 1.** Demographics of individual cases completing the study.

| Case | Dose  (mg CBD /kg bw) | Breed | Weight  (kg) | Age  (y) | Sex | BCS  (/9) | Affected limb | Complete or Partial Tear | Meniscal Tear (yes/no) | Bilateral CCLr (yes/no) |
| --- | --- | --- | --- | --- | --- | --- | --- | --- | --- | --- |
| 1 | 5 | Labrador X | 23.2 | 10 | F(S) | 5 | R | P | N | N |
| 2 | 2 | Bulldog | 42.2 | 4 | M(N) | 6 | L | P | Y | N |
| 3 | 0 | GSD X | 24.5 | 10 | F(S) | 5 | R | N/D | Y | N |
| 4 | 5 | Labrador | 30 | 4 | F(S) | 6 | R | P | N | N |
| 5^1^ | 2 | GSD X | 35.9 | 7 | M(N) | 5 | R | P | Y | N |
| 6 | 2 | Blue Heeler | 22.1 | 7 | M(N) | 6 | R | N/D | Y | N |
| 7 | 5 | GSD X | 32 | 7 | F(S) | 7 | L | P | Y | N |
| 8^2^ | 0 | Husky X | 32.5 | 4 | F(S) | 6 | L | P | Y | Y |
| 9 | 2 | Bulldog | 36 | 5 | F(S) | 8 | R | C | Y | N |
| 10 | 5 | Mix | 36 | 7 | M(N) | 7 | R | P | Y | N |
| 11^3^ | 0 | GSD X | 43.6 | 5 | M(I) | 6 | L | C | N | N |
| 12 | 2 | Husky X | 37.9 | 5 | M(N) | 7 | L | P | Y | Y |
| 13 | 5 | Mix | 35 | 4 | M(N) | 5 | L | C | Y | N |
| 14 | 0 | GSD X | 23.5 | 11 | F(S) | 5 | L | C | Y | N |
| 15 | 2 | GSD X | 52 | 4 | M(N) | 6 | R | P | Y | N |
| 16 | 0 | Staffordshire Terrier | 24.6 | 6 | M(N) | 8 | R | C | Y | Y |
| 17 | 2 | GSD | 42 | 8 | M(N) | 6 | R | C | Y | N |
| 18^2^ | 5 | Husky X | 30 | 5 | F(S) | 5 | L | P | N | N |
| 19 | 0 | Labrador | 27 | 3 | F(S) | 8 | L | C | Y | N |
| 20 | 5 | Labrador X | 35 | 4 | M(N) | 6 | L | P | N | Y |
| 21 | 0 | Australian Shepherd | 31.1 | 8 | M(N) | 8 | R | C | Y | N |
| 22 | 2 | Doberman X | 29.3 | 6 | F(S) | 6 | L | N/D | Y | N |
| 23 | 5 | Border Collie | 30.2 | 7 | M(I) | 7 | L | N/D | Y | Y |
| 24^4^ | 2 | Rottweiler X | 35 | 8 | F(S) | 6 | R | N/D | N | N |
| 25 | 0 | Labrador X | 31.8 | 8 | M(N) | 7 | L | C | Y | Y |
| 26 | 2 | GSD X | 36.6 | 8 | F(S) | 6 | R | C | N | N |
| 27^3^ | 2 | GSD X | 44 | 5 | M(I) | 7 | R | P | Y | N |
| 28 | 0 | Labrador | 31.8 | 6 | M(N) | 6 | R | P | Y | N |
| 29 | 0 | Rottweiler | 32 | 4 | F(S) | 6 | R | C | Y | N |
| 30 | 2 | RSD X | 44.2 | 9 | F(S) | 7 | L | N/D | Y | N |
| 31^5^ | 5 | Labrador X | 22.3 | 4 | F(S) | 6 | R | P | N | Y |
| 32 | 5 | Weimaraner | 36.1 | 6 | F(S) | 5 | L | C | N | N |
| 33 | 5 | Husky X | 26.1 | 5 | F(S) | 5 | R | P | N | Y |
| 34 | 0 | Husky | 15.4 | 9 | F(I) | 5 | R | C | N | Y |
| 35 | 2 | Labrador X | 30.8 | 4 | F(S) | 7 | R | C | Y | N |
| 36 | 5 | GSD X | 22.7 | 5 | F(S) | 5 | L | P | N | N |
| 37^4^ | 0 | Rottweiler X | 31 | 9 | F(S) | 6 | L | P | N | N |
| 38^5^ | 0 | Labrador X | 21.5 | 4 | F(S) | 5 | L | C | Y | N |
| 39 | 5 | Boxer X | 37.6 | 11 | M(N) | 5 | L | C | Y | N |
| 40 | 0 | Pitbull X | 26.5 | 4 | F(S) | 4 | R | N/D | N | Y |
| 41^1^ | 0 | GSD X | 34.3 | 8 | M(I) | 5 | L | C | N | N |
| 42 | 0 | Bernese Mountain Dog | 35 | 9 | F(S) | 6 | L | C | Y | N |

BCS = Body Condition Score on 1–9 scale

N/D = not determined

GSD = German Shepherd Dog

Numerical superscripts indicate the same dog presented for multiple TPLO surgeries.

Affected limb notes whether the TPLO surgery was performed on the left or right hind limb.

Bilateral CCLr indicates whether cases that had (uncorrected) cranial cruciate ligament ruptures on both stifles at the time of TPLO surgery. Dogs returning for TPLO surgery on the second limb were classified as “unilateral” for that surgery, due to previous TPLO correction of the first limb.

**Supplemental Table 2**. Plasma Concentration (ng/mL) of CBD and THC in individual dogs on day +1 and day +14.

| Dose | Case | CBD D+1  (ng/mL) | CBD D+14  (ng/mL) | THC D+1  (ng/mL) | THC D+14  (ng/mL) |
| --- | --- | --- | --- | --- | --- |
| Placebo  (n=8) | 3 | < LOD | n/a | <LOD | n/a |
|  | 8 | < LOD | < LOD | < LOD | < LOD |
|  | 11 | < LOD | < LOD | < LOD | < LOD |
|  | 14 | < LOD | n/a | < LOD | n/a |
|  | 16 | < LOD | n/a | < LOD | n/a |
|  | 19 | < LOD | < LOD | < LOD | < LOD |
|  | 34 | < LOD | < LOD | < LOD | < LOD |
|  | 37 | < LOD | < LOD | < LOD | < LOD |
| Low dose CHE  (n=9) | 2 | 177 | n/a | 14.5 | n/a |
|  | 5 | 130 | n/a | 11.4 | n/a |
|  | 6 | 2.6 | 106 | < LOD | 4.2 |
|  | 9 | 42 | 107 | 3.84 | 5.39 |
|  | 12 | <LOQ | 383 | < 0 | 32.9 |
|  | 15 | <LOD | n/a | < LOD | n/a |
|  | 17 | 10.2 | 53.7 | 0.373 | 3.04 |
|  | 30 | 274 | 271 | 58 | 40.7 |
|  | 35 | 18.5 | n/a | 4.21 | n/a |
| High dose CHE  (n=9) | 1 | 109 | n/a | 9.72 | n/a |
|  | 4 | 135 | 415 | 8.22 | 9.17 |
|  | 7 | <LOQ | 426 | <LOD | 12.8 |
|  | 10 | 51.3 | 80.6 | 2.97 | 3.11 |
|  | 13 | 566 | n/a | 47.3 | n/a |
|  | 20 | 123 | 56.2 | 12.2 | 2.75 |
|  | 31 | 151 | 205 | 39.5 | 28.7 |
|  | 32 | 33 | 761 | 7.48 | 86 |
|  | 33 | 23.9 | 115 | 8.1 | 19.4 |

Low dose CHE = 2 mg CBD +0.1 mg THC / kg bw

High dose CHE = 5 mg CBD + 0.25 mg THC / kg bw

Limit of quantification (LOQ) = 2.0 ng/mL.

Limit of detection (LOD) = 0.5 ng/mL

n/a = uncollected samples (due to lack of D+14 recheck, no dose administered within 24 hours of clinic visit, or sample not taken due to aggressive dog).

**Supplemental Table 3**. Plasma concentration (ng/mL) of select CBD and THC metabolites in individual dogs on day +1 and day +14.

| Case | CHE Dose | 6-OH-CBD  (ng/mL) | | 7-OH-CBD  (ng/mL) | | 7-COOH-CBD  (ng/mL) | | 11-OH-THC  (ng/mL) | | 11-COOH-THC  (ng/mL) | |
| --- | --- | --- | --- | --- | --- | --- | --- | --- | --- | --- | --- |
|  |  | Day +1 | Day +14 | Day +1 | Day +14 | Day +1 | Day +14 | Day +1 | Day +14 | Day +1 | Day  +14 |
| 30 | Low | 39.4 | 79.5 | 8.3 | 11.3 | 28 | 26.4 | 1.42 | 4.75 | <LOD | 0.665 |
| 31 | High | 15.6 | 52.8 | 2.3 | 5.68 | 20.6 | 21.8 | <LOQ | 3.82 | <LOD | <LOD |
| 32 | High | 111 | 5.08 | 34.8 | 2.26 | 46.7 | 3.95 | 10.6 | <LOD | 0.887 | <LOD |
| 33 | High | 19.2 | 2.9 | <LOQ | <LOQ | 10.5 | 2.48 | 2.31 | <LOD | <LOD | <LOD |
| 35 | Low | <LOQ | n/a | <LOD | n/a | <LOQ | n/a | <LOD | n/a | <LOD | n/a |

Low dose CHE = 2 mg CBD +0.1 mg THC / kg bw

High dose CHE = 5 mg CBD + 0.25 mg THC / kg bw

Limit of quantification (LOQ) = 2.0 ng/mL.

Limit of detection (LOD) = 0.5 ng/mL

n/a = uncollected sample
